# Supplementary material for: Botulinum Toxin for Axial Postural Abnormalities in Parkinson’s Disease: A Systematic Review
Source: Toxins (Basel). 2024 May 15;16(5):228. doi: 10.3390/toxins16050228 (PMC11125648; doi:10.3390/toxins16050228)
Supplement: Supplementary file 1 [file toxins-16-00228-s001.zip › toxins-2946652-supplementary.pdf]

toxins MDPI

Table S1. Detailed information of studies evaluating the effects of BoNT A (Group A) and BoNT A combined with physical rehabilitation (Group B) for treating APAs in PD.

Study ID<br>Type of PA<br>Study design	No. of patients (gender)<br>Age at evaluation<br>PD duration	PA duration<br>Diagnostic criteria of PA<br>Severity of PA, if reported	Type of Toxin<br>Injection method<br>N of injection cycles, if reported	Muscles injected	Study outcomes measures and follow-up (if reported)	Main Results	QA-LE
**Group A. Studies evaluating the only effect of BoNT A for APAs in PD**							
Yahalom et al, 2023 [82]<br>AC<br>Case series	1 (M)<br>-<br>76 y<br>-<br>3 y	More than 3 y (requiring surgery for myelopathy)<br>–<br>n.r<br>-<br>Marked antecollis, bilateral increased tonus of the trapezius pars descendens and of the scalene muscles, without weakness of the cervical extensor muscles	Abobotulinum (Dysport)<br>Method: n.r.	Bilateral Medial Scalene (50 U)<br>Bilateral Levator Scapulae (25 U)	Neurological examination	Absence of improvement (objective or subjective) of antecollis after BoNT	Poor - 4
Seliverstov et al. 2020 [89]<br>AC	2 pts<br>Pt 1 (F) - 66 y - 11 y	Pt 1: 1 y, Antecollis with mild laterocollis	Onabotulinum (Botox)<br>EMG-guided,	Pt 1:<br>- Longus Colli: 50 U<br>- Anterior Scalene: 10	Duration of the Improvement	Pt 1 4-week follow-up:<br>- Reduction of neck pain in anterior region and enhanced	Fair - 4

Case series	Pt 2 (M) - 63 y - 8 y	Pt 2: 5 m<br>-Variability of the posture as well as significant regression in a supine position<br>- Limiting dressing, mobility, and maintenance of social interactions	patient supine	U on each side<br>- Levator Scapulae: 15 U/side<br><br>Pt 2:<br>- Longus colli 40 U/side	Satisfaction rate<br><br>Side effects	capability to sustain a more upright posture of the head-Satisfaction rate 40%<br>- Side effect: mild dysphagia for 1 week<br><br>Pt 2 4-week follow-up:<br>- Improved neck mobility and communication abilities<br>- Satisfaction rate of 30%<br>- Side effects: difficulties in maintaining an upright neck posture; moderate dysphagia for 1.5 months	
Van de Warrenburg et al, 2007 [83]<br><br>AC<br><br>Case series	n=9 PD (3F,6M)<br>-<br>69.1 ± 5.8 y<br>-<br>10.5 ± 7 y	2.7 ± 1.4 y<br>-<br>Forward flexion and anterior shift of the neck with prominent cervical paraspinal and levator scapulae muscles, without residual neck extension weakness	Abobotulinum (Dysport): n=7 pt<br><br>Method: EMG prior to injection	Uni/bilateral Levator scapulae: 50-250 U<br><br>Uni/bilateral Sternocleidomastoid 50-100 U<br><br>Uni/bilateral Splenius capitis 200 U	Pain<br><br>Neurological examination	The majority of patients did not experience benefits from these injection .<br><br>Pain reduction (1 pt)<br><br>Improvement of the AC (2 pts)	Poor – 4

Matsumoto et al., 2018 [90]<br><br>CC+PS<br><br>Case report	1 (M)<br>-<br>64 y<br>-<br>6 y	5 y<br>-<br>Anterior trunk flexion<br>LTF (right side)<br>-<br>Severe right abdominal pain requiring access to emergency room	Onabotulinum (Botox): n=1 patient<br><br>Method: palpation of hypertrophic muscle<br><br>Two cycles, one month apart	Ipsilateral paraspinal (right) muscles from L2 to L4 spinous processes level<br><br>First session 20 U<br>Second session 30 U	Pain<br>Postural improvement by body X-ray and Surface EMG	Pain relieved<br><br>Improvement of posture at body X-ray<br><br>Increased contralateral tonic contraction of lumbar paraspinal muscles; decreased in tonic contraction of ipsilateral lumbar paraspinal muscles by surface EMG analysis.	Fair - 4
Todo et al., 2018 [84]<br><br>CC<br><br>Case series	6 (4F, 2M)<br>-<br>72.5 ± 8.5<br>-<br>10.6 ± 5.3 y	2.3 ± 0.8<br>-<br>Thoracic spine flexion.<br>-<br>Severe symptoms: Abdominal contractions (2 pts), dysbasia (1 pts), difficulty in facing upward (3 pts)	Onabotulinum (Botox)<br><br>Method: US guidance, two injections at the intersection of the anterior axillary line and the line level with the umbilicus	Bilateral External Abdominal Oblique muscle (range): 75 to 90 U into each side.<br><br>100-120 U into each side as maintenance dose (every 3-4 months, 3 patients)	Short term evaluation at 2 weeks and long term (1 year or more):<br>- CC degree before vs. after BoNT<br>- EMG findings<br>- VAS	Significant mean angle of CC improvement from median of 38° (interquartile: 23.5°) to 18° (interquartile:21°).<br><br>4/6 pts reported subjective relief.<br><br>VAS of 2 pts with abdominal contraction from 84 and 98 to 4 and 77<br><br>Long-term: two cases with persistent benefit, one reduced	Poor - 4

Wijemanne et al., 2014 [94]<br><br>CC<br><br>Case report	1 (F)<br>-<br>66 y<br>-<br>7 y	2 y (and history of left Transverse Rectus Abdominis Myocutaneous flap procedure for breast reconstruction)<br>-<br>Palpable abdominal contractions in standing position.<br>Pt was unable to lie flat on her back and had to lean upright against a wall.<br>-<br>Painful abdominal contractions with a 45-degree forward spine flexion in the OFF	Onabotulinum (Botox)<br><br>Method: EMG-guided<br><br>Two cycles, at a distance of 8 weeks Then, every 3-4 months	First injection: 200 U for each Rectus Abdominis<br><br>Second injection: 200 U in left External Abdominal Oblique 200 U in right Rectus Abdominis	Anterior trunk flexion degree over 18 months	CC maintained a 15-20° improvement in the 'ON' state with a UPDRS score of 8, compared to 30° in the 'OFF' state, showing good response over 18 months	NA - 4
Colosimo et al., 2009 [86]<br><br>CC<br><br>Case series	2 (n.r).<br>-<br>n.r	n.r<br>-<br>Reducible forward flexion of the thoracolumbar spine > 45°<br>-<br>Severe CC	Onabotulinum (Botox)<br><br>Method: CT guidance	300 U for each Iliopsoas (deep lumbar portion):<br><br>200 U for each Rectus Abdominis	Clinical evaluation	No objectively or subjective improvement of CC at 1 day, 1 week, and 2 weeks after BoNT injection	Poor - 4
Fietzek et al., 2009 [85]<br><br>CC	10 (M/F n.r.)<br>n=5 (age 70 ± 4.5	Iliopsoas group (1.9 ± 0.2), Rectus abdominis	Incobotulinum (Xeomin)	50 U per site<br><br>Bilateral Iliopsoas (4-	After 3 weeks:	No improvement	Poor - 4

Goal attainment controlled study	y) injected in the Iliopsoas<br><br>n= 5 (age 75.4 ± 1.6 y) injected in the rectus abdominis)	group (3.0 ± 1.4)<br>-<br>n.r.<br>-<br>Treatment goals included achieving a more upright gait (N=6), pain relief (N=3), enabling the grasping of previously unreachable items (N=3), facilitating social activities (N=2), and reducing stigmatization (N=3)	Method: US guidance	6 sites) muscle injected directly below the inguinal ligament with two to three sites (mean dosage 210 ± 50 U)<br><br>Bilateral Rectus Abdominis (6-8 sites) (mean 200 ± 63 U)	- Timed Up&Go 10 m<br>- Goal attainment		
Von Coelln et al., 2008 [91]<br><br>CC<br><br>Case series	3 (M)<br>-<br>75y, 73y, 70y<br>-<br>16y, 8y, 10y	3y, 1y, 1.5y<br>- Abnormal trunk posture with marked thoracolumbar spine flexion when standing and walking, which lessens in the supine position	Abobotulinum (Dysport)<br><br>Method: US guidance	Unilateral, ipsilateral Psoas Major (n=2 PD with trunk deviation forward and toward the right side); Bilateral Psoas Major involvement (n=1 PD with pure forward flexion), treated via a ventral approach	Evaluation at 2, 4, and 16 weeks after each treatment:<br>- Detailed interview<br>- Physical examination<br>- Body height (spontaneous and in maximal effort to stand)	All pts complained a mild to moderate weakness of hip flexion at the highest dose of BoNT.<br><br>Limited or no efficacy in all patients	Fair - 4

				above the inguinal ligament, along the muscle's longitudinal axis<br>Initial dose: 500 U/side<br><br>Retreatment every 4-6 months with a total of 16 injections, dosage increased by 500 U to a maximum of 1500 U per side			
Azher et al., 2005 [35]<br><br>CC<br><br>Case series	6 (4M, 2F)<br>-<br>67.2 ± 6 y<br>-<br>12 ± 7.2 y	3.8 ± 2.1 y<br>-<br>A significant (>45°) flexion of the thoracolumbar spine that intensifies during walking and substantially decreases or vanishes in the recumbent position	Onabotulinum (Botox)<br><br>Method: n.r.	Rectus Abdominis (4 pts, range 150-400 U)<br>Paraspinal muscles (2 pts, 200 U)<br>Paraspinal muscles (2 pts, unknown dosage)	Clinical effect and its duration	Good response in 3 pts (8 weeks, 14 weeks, 24 weeks)<br><br>Not reports of 3 pts	Poor - 4
Ledda et al 2023 [92]<br><br>PS	13 (8 M, 5 F)<br>-<br>68.8 ± 9<br>-	4.1 ± 5<br>-<br>PS: >10 degrees of LTF, manifests	Onabotulinum (Botox)<br>-<br>Method: US guidance	1st injection: 50 U longissimus thoracis and 50 U	Short -term (4 months) and	Short-term (13 pts):<br>No improvement of angle of LTF and VAS	Fair - 4

Longitudinal observational study and case-control	12.7 ± 6.2<br><br>Control pts (untreated):<br>7 PD pts with PS (4 M, 3 F)<br>-<br>69.7 ± 3.2<br>-<br>7.7 ± 5.8	when sitting or standing .<br><br>LTF: involuntary lateral trunk bending, 5 to 10 degrees-<br>Baseline standing flexion angle: 11.2 ± 4.6 degrees; back pain VAS score: 6 ± 3<br>Controls baseline angle of flexion 15.1 ± 4.3	EMG:<br>a) Seated without upper limb or trunk support.<br>b) With trunk held upright and straight by an operator<br>c) during patient's self-realignment of the trunk.<br><br>4 BoNT cycles, administered every 4 months	iliocostalis lumborum ipsilateral to LTF<br><br>From 2nd injection: personalized treatment based on US and EMG (longissimus thoracis and/or iliocostalis lumborum muscles with pathological muscular hyperactivity, from 15 to 50 units for each injected muscle)	Long-term (12 months):<br>- Posture assessment (NeuroPosture APP)<br>- VAS score	Long-term (7 pts): Mild improvement of angle of LTF (from 10.4 ± 2.8 to 9.2 ± 6.4 degrees)<br><br>Angle of LTF in controls at long-term (18 months): 20.2 ± 7.4 degrees<br><br>Significant differences in LTF degree changes over time between treated and untreated control groups (p = 0.026)	
Artusi et al., 2019 [88]<br><br>PS<br><br>Prospective, pilot study	13 (10M, 3F)<br>-<br>70.5 ± 8.5 y<br>-<br>Age at PD onset 60.0 ± 12.0 y	2.7 ± 2.3 y -<br><br>LTF ≥ 10° improved by passive mobilization and supine positioning.	Onabotulinum (Botox)<br><br>MRI to assess atrophy of paraspinal and non-paraspinal axial muscles<br><br>Method: US and EMG-guided.<br>EMG was	Muscle selection: hyperactivity on EMG when standing (if not available, based on lying findings); absence of severe muscle atrophy on MRI.	Postural improvement observed two months after BoNT injection.<br><br>Degrees of LTF changes measured using a wall goniometer and "ImageJ," with a 5°	The rate of responders was 84.6% (n= 11/13)<br><br>LTF angle improved by 40%, from 15.7 ± 8.4 to 9.4 ± 11.8 degrees.<br><br>Pain reduction was 52.2% in total VAS score, from 6.9 ± 2.2 to 3.3 ± 1.6	Good – 2c

			conducted in lying down prone on an examination table with arms along the trunk, and then in standing position	Paraspinal muscles at L2-L4 (longissimus thoracis, iliocostalis lumborum, and spinalis dorsi) (range: 50 - 75 U)<br><br>Non paraspinal muscles (abdominal oblique and Iliopsoas) (range 25 to 50 U)	cut-off defining significant improvement.<br><br>VAS for procedure-related adverse events and side effects (AEs)	No procedural or post-procedural adverse events (AEs)	
Dupeyron et al., 2015 [95]<br><br>PS<br><br>Case report	1 (F)<br>-<br>69 y<br>-<br>10 y	A tonic LTF toward one side with complete resolution in supine position and increased deviation during standing	Incobotulinum (Xeomin)<br><br>EMG before injection	First injection: 50 U in two sites (T10 and L3 level), of left iliocostalis (Ipsilateral) muscle<br><br>Second injection, three months after: 50 U in two sites of ipsilateral quadratus	Clinical improvement with pictures	Six weeks after first injection pain improved, but not PS.<br><br>Improvement of PS after second injection, lasting for one year	NA<br>- 4

				lumborum muscle			
Bonanni et al., 2007 [93]<br><br>PS<br><br>Blinded cross-over trial	9 (5M,4F)<br>n=4 BoNT treatment<br><br>n=5 placebo<br>-<br>71.8 ± 5.4 y<br>-<br>10.3 ± 7.2 y	2.4 ± 0.9 y<br>-<br>LFT ≥ 15° that increase during walking and disappear in the recumbent position.<br><br>LTF is defined by continuous EMG activity in the lumbar paraspinal muscles on the same side as the bend, occurring only when the patient is standing or walking.<br>Baseline VAS 68.3 ± 11.3<br>Baseline TDSS 7 ± 2.8<br>Baseline bending degrees 30.6 ± 6.9	Abobotulinum (Dysport): 500 U<br>-<br>Method: EMG-guided (to evaluate continuous EMG activity of lumbar paraspinal muscles ipsilateral to the bending side, appearing only when standing or walking, disappearing in lateral recumbent position<br>-<br>Following the blinded evaluation, BoNT injections were repeated quarterly over a two-year period	Injection of 125 U into four sites of paraspinal muscles, 2 to 2.5 cm lateral to the spinous processes at levels L2-L5, on the side ipsilateral to the bend	Evaluation at 2 and 4 weeks, and at 3 months:<br>- TDDS<br>- VAS<br>- Goniometric measurement of the LTF displacement	At 1 month, n=6 pts showed an improvement of lateral bending from 50% to 85.7% and reduction of pain<br><br>Pts treated with BoNT showed an average improvement of 4 points on the TDDS score and a 31.4 mm average reduction in pain, with improvements ranging from 24.1% to 76.4% on the VAS scale from baseline	Fair - 4
**Group B. Studies of BoNT combined with physical rehabilitation for APAs in PD**							

Tassorelli et al., 2014 [87]<br><br>PS+CC<br><br>RCT	26 patients<br>-<br>n=13 group A treatment group (8F, 5M)<br><br>n=13 group B placebo (6F, 7M)<br>-<br>Group A 73.9 ± 4.3 y<br>Group B 74.2 ± 5.1 y<br>-<br>Group A 10.2 ± 8.2 y,<br>Group B 10.4 ± 10.1 y	Group A: 3.1 ± 1.9 y<br>Group B: 3.0 ± 1.5.<br>-<br>PS: mild to moderate LTF (Cobb's angle >10°),<br>CC: static anterior trunk flexion<br>-<br>VAS group A = 6.7 ± 1.5<br>VAS group B = 5.4 ± 2.5	Incobotulinum (Xeomin)<br>-<br>Method EMG-guided (injection if involuntary tonic activity longer than 500 ms)<br>-<br>Rehab: 5 days/week for 4 weeks	Maximum 6 sites per patient, with a maximum dose of 50 UI of BoNT per site, with a total dose per patient ranging between 50 to 200 UI.<br><br>Bilateral Iliopsoas, ipsilateral or contralateral Multifidus, Rectus Abdominis, inferior thoracic and lumbar paravertebral muscles	Evaluation at the end of rehab, and 3 and 6 months after.<br>- VAS of back pain<br>- UPDRS score<br>- FIM score<br>- Degree of anterior trunk flexion<br>- trunk ROM on the 4 plans<br>- Kinematic analysis<br>- Lateral inclination and anterior flexion while standing	Groups A and B improved significantly in static postural alignment and ROM at the end of the treatment period (BoNT/saline + rehabilitation)..<br><br>Group A exhibited a significantly more reduction in pain scores compared to Group B, and also a longer-lasting efficacy on several clinical and kinematic variables.	Good – 1b
Santamato et al., 2010 [96]<br><br>PS<br><br>Case report	1 M<br><br>n.r.	n.r.-<br>Significant deviation of the trunk to the right	Abobotulinum (Dysport)<br><br>Method: EMG recordings + multidisciplinary rehabilitation program	Bilateral paraspinous muscles (2–2.5 cm lateral to the spinous processes, T10-L2)<br><br>600 U distributed across 6	LTF angle<br>TDDS<br>VAS	15 days after: improvement of PS from 35° to 15°, improvement of 5 points in TDDS, and a reduction of VAS score from 7 to 3<br><br>After 3 months, the improvement	NA - 4

	Rehab: 5 days/week, After 3 months reduced to 3days/week	injection sites (100 U per site)		appeared to be maintained

Abbreviations: APAs, Axial Postural Abnormalities; BoNT, Botulinum toxin; CC, Camptocormia; CT, Computed tomography; EMG, Electromyography; US, ultrasound; M, Male; F, Female; FIM, Functional Independence Measure; LE, Levels of Evidence; LTF, Lateral trunk flexion; ms, milliseconds; PD, Parkinson's disease; PS, Pisa syndrome; AC, Antecollis; Pt, patient; ROM, range of motion; SD, Standard deviation; U, units; QA, Quality of Assessment; RCT, Randomized Controlled Trial; TDDS, Trunk Dystonia Disability Scale; UPDRS, Unified Parkinson's Disease Rating Scale; US, Ultrasound; VAS: Visual Analogue Scale; y, years; NA, not available; n.r., not reported.

Supplementary S1

This section provides detailed information on the search strategy for all databases. Pubmed

(Pisa syndrome OR lateral trunk flexion OR Camptocormia OR Anterior trunk flexion OR Forward trunk flexion OR Retrocollis OR Anterocollis OR Stooped posture OR Spinal curvatures OR Antecollis OR Cervical Dystonia OR torticollis) AND (Parkinsonian disorders OR Parkinson Disease OR Multiple System Atrophy OR Progressive Supranuclear Palsy OR Dementia OR Lewy body OR Corticobasal syndrome OR Axial manifestation OR Postural syndrome)) AND ("Surgical Procedures, Operative"[Mesh]) OR ("Orthopedic Equipment"[Mesh] OR corset) OR ("Chemicals and Drugs Category"[Mesh] OR drug) OR (treatment OR "Therapeutics"[Mesh] OR Therapy OR rehabilitation) AND (outcome OR evaluation OR effect OR result OR efficacy OR assessment) AND (severity OR posture OR walking OR postural OR balance OR equilibrium OR gait OR deambulation)

Web of Science All databases

("Parkinsonian disorders" OR "Parkinson Disease" OR "Multiple System Atrophy" OR "Progressive Supranuclear Palsy" OR Dementia OR "Lewy body" OR "Corticobasal syndrome" OR "Axial manifestation" OR "Postural syndrome") AND ("Pisa syndrome" OR "lateral trunk flexion" OR Camptocormia OR "Anterior trunk flexion" OR "Forward trunk flexion" OR Retrocollis OR Anterocollis OR "Stooped posture" OR "Spinal curvatures" OR Antecollis OR "Cervical Dystonia" OR torticollis) AND (outcome OR evaluation OR effect OR result OR efficacy OR assessment OR evaluate OR assess) AND (severity OR posture OR walking OR postural OR balance OR equilibrium OR gait OR deambulation) AND (surg* OR drug OR therap* OR rehabilit* OR stimulation OR exercise OR manipulation OR ortho* OR corset OR walker OR crunches OR lidocaine OR pallidotomy)

SCOPUS

("Parkinsonian disorders" OR "Parkinson Disease" OR "Multiple System Atrophy" OR "Progressive Supranuclear Palsy" OR Dementia OR "Lewy body" OR "Corticobasal syndrome" OR "Axial manifestation" OR "Postural syndrome") AND ("Pisa syndrome" OR "lateral trunk flexion" OR Camptocormia OR "Anterior trunk flexion" OR "Forward trunk flexion" OR Retrocollis OR Anterocollis OR "Stooped posture" OR "Spinal curvatures" OR Antecollis OR

"Cervical Dystonia" OR torticollis) AND (outcome OR evaluation OR effect OR result OR efficacy OR assessment OR evaluate OR assess) AND (severity OR posture OR walking OR postural OR balance OR equilibrium OR gait OR deambulation) AND (surg* OR drug OR therap* OR rehabilit* OR stimulation OR exercise OR manipulation OR ortho* OR corset OR walker OR crunches OR lidocaine OR pallidotomy)
